# An Association between Insulin Resistance and Neurodegeneration in Zebrafish Larval Model (*Danio rerio*)

**DOI:** 10.3390/ijms23158290

**Published:** 2022-07-27

**Authors:** Nurliyana Najwa Md Razip, Suzita Mohd Noor, Anwar Norazit, Norshariza Nordin, Nurshafika Mohd Sakeh, Huzwah Khaza’ai

**Affiliations:** 1 Department of Biomedical Sciences, Faculty of Medicine and Health Sciences, Universiti Putra Malaysia, Serdang 43400, Selangor, Malaysia; najwabiochem@gmail.com (N.N.M.R.); shariza@upm.edu.my (N.N.); 2 Department of Biomedical Science, Faculty of Medicine, University of Malaya, Kuala Lumpur 50603, Selangor, Malaysia; suzita@um.edu.my (S.M.N.); anwar.norazit@um.edu.my (A.N.); 3 Genetics & Regenerative Medicine (ReGEN) Research Group, Faculty of Medicine and Health Sciences, Universiti Putra Malaysia, Serdang 43400, Selangor, Malaysia; 4 Apta Niaga Sdn. Bhd, No. 12, Jalan Desa Melawati 1A, Desa Melawati, Kuala Lumpur 53100, Selangor, Malaysia; aptaniaga@outlook.com

**Keywords:** glucose, insulin resistance, neurodegeneration, type 2 diabetes mellitus, zebrafish

## Abstract

Background: Type 2 diabetes mellitus has recently been identified as a mediator of neurodegeneration. However, the molecular mechanisms have not been clearly elucidated. We aimed to investigate insulin resistance associated with neurodegenerative events in zebrafish larvae. Methods: Larvae aged 72 h-post-fertilization (hpf) were induced to insulin resistance by immersion in 250 nM insulin and were then reinduced with 100 nM insulin at 96 hpf. This model was validated by a glucose levels assay, qPCR analysis of selected genes (*akt*, *pepck*, *zglut3* and *claudin-5a*) and Oil Red-O (ORO) staining of the yolk sac for lipid distribution. The association of insulin resistance and neurodegeneration was validated by malondialdehyde (MDA), glutathione (GSH) assays, and by integrating next-generation sequencing with database for annotation, visualization and integrated discovery (DAVID). Results: There was a significant increase in glucose levels at 180 min in the insulin-resistant group. However, it decreased at 400 min after the re-challenge. Insulin-signaling mediators, *akt* and *pepck,* were showed significantly downregulated up to 400 min after insulin immersion (*p* < 0.05). Meanwhile, *claudin-5a* assessed blood–brain barrier (BBB) integrity and showed significant deterioration after 400 min of post-insulin immersion. ORO staining remarked the increase in yolk sac size in the insulin-resistant group. After the confirmation of insulin resistance, MDA levels increased significantly in the insulin-resistant group compared to the control group in the following parameters. Furthermore, dysregulated MAPK- and Wnt/Ca^2+^-signaling pathways were observed in the insulin-resistant group, disrupting energy metabolism and causing BBB injury. Conclusions: We conclude that the insulin-resistant zebrafish larvae alter the metabolic physiology associated with neurodegeneration.

## 1. Introduction

Insulin resistance in type 2 diabetes mellitus (T2DM) is a devastating disease that has a global prevalence of more than 400 million people and is expected to increase by 9% by 2045 [1]. In Malaysia, it contributes to a high diabetes healthcare burden in Asia [2]. The pathophysiological changes in T2DM such as insulin resistance, hyperglycemia and beta-cell dysfunction may pathologically cause elevation in inflammation, oxidative stress, mitochondria destruction and lipotoxicity [3,4,5]. T2DM is thought to induce neuropathology such as in central nervous system (CNS) and brain, which results in the complication of cognitive dysfunction and neurodegeneration [6,7]. The associations of T2DM and brain function depletion are recognized between peripheral insulin resistance overlapping with the neuropathology from the tau protein deposits and amyloid beta plaque accumulation in the brain anatomy [8]. However, the targeted mechanism pathways from the beginning of hepatic-insulin-resistance development to causing neurodegeneration are not clearly understood. The underlying cascade response, nevertheless, is still being investigated.

Focusing on the molecular mechanism of insulin cascade signaling prior to the T2DM pathophysiology is crucial to investigate the neurodegeneration consequences. Kim et al. (2011) reported that insulin resistance in the cortical neurons of diabetic mice perturbed phosphatidylinositol-3-kinase (PI3K) signaling and reduced neuroprotection [9]. Even more, in a current study, the neuronal SK-N-SH cell line induced with hyperinsulin at 250 nM was found to downregulate insulin cascade via the Akt dephosphorylation and activation of GSK3beta that enhance the overproduction of tau protein [10]. Evidently, underlying glucose homeostasis dysregulation in neuronal cells promotes cognitive deficits.

The alteration of glucose metabolism from T2DM may impede the neuronal function, causing apoptosis and eventually resulting in neurodegeneration, particularly in dementia or Alzheimer’s disease (AD) [11]. In CNS, on the other hand, Akt protects from amyloid beta toxicity [12,13,14], tau homeostasis [15], neuroinflammation [16] and neurofibrillary tangles formation [10,17]. The PI3K/Akt-signaling pathway is a key mediator for the cell growth, energy production and cell proliferation that has importance in brain cognition [18]. It begins with insulin receptors binding with the insulin hormone, triggering the cascade of lipid kinase activation and phosphorylation. Consequently, phosphatidylinositol triphosphate (PIP3) synthesizes and recruits the phosphoinositide-dependent kinase (PDK) directly to phosphorylate threonine-308 residue of Akt, resulting in the activation of the mitogen-activated protein kinase (MAPK)-signaling pathway [18]. The activation of PI3K by Akt also releases glucose transport-4 (Glut4) into the vesicle by exocytosis to the plasma membrane to enhance the glucose uptake velocity into the cells [19]. These effector molecules mediate the effects of insulin on glucose production, utilization and uptake that cause Glut4 to ensure its role in taking up the glucose into the intracellular membrane [20]. Glucose undergoes glycolysis and Kreb’s cycle, producing energy and other metabolites that serve as precursors of fatty acid, lipid and protein metabolism [21]. It is hypothesized that insulin resistance in T2DM could increase the risk of neurodegeneration. Multiple downstream chain reactions in metabolic disorders modulate cell starvation and promote oxidative stress. Lipogenesis is another event that occurs due to insulin resistance [22]. In this study, the yolk of zebrafish larvae will be used to assess the lipid distribution measurement.

The multifaceted mechanisms in T2DM are difficult to grasp; however, with the zebrafish as an animal model, the projection of complex organisms can be well understood. A genetic study in zebrafish has been defined in more than 70% of the human genome and mammal [23]. The physiology of glucose homeostasis has been preserved in zebrafish, to fulfill the understanding of the disease model correlated to humans. As mentioned earlier, the endocrine system of zebrafish plays a role in stimulating the insulin signaling mechanism and development of fish. The beta-cell mass production of insulin from the endocrine pancreas is anatomically embedded within the exocrine pancreas [24]. Previous studies used a zebrafish model to study how hyperinsulinemia-induced insulin resistance associated with immune response exhibited the inhibition of key mediators of the insulin-signaling pathway. Coinciding with the loss of insulin sensitivity, perturbation of glucose homeostasis was observed due to the downregulation of phosphoenolpyruvate carboxykinase (*pepck*) and the dephosphorylation of the Akt pathway upon microinjection of insulin (250 nM and 100 nM) into the caudal aorta at 4 days post fertilization (dpf) in larvae [25]. Similarly, Rocha et al., (2013) and Nam et al., (2021) studies indicate that glucose acts on the physiological changes in zebrafish larvae by glucose and insulin immersion, respectively [26,27]. Nam et al., (2021) focused on the characterization of insulin resistance in a T2DM model in MAPK signaling pathways encoded by the *map2k6*-gene-activated inflammatory cytokines [27]. However, the association with T2DM-targeted neurodegeneration pathways has yet to be proven.

Orthologue genes in the insulin signaling pathway are expressed in zebrafish such as glucose transporter (Glut) genes *Glut-1*, *2*, *3*, *10* and *12* [25,26,27,28,29,30]. The expression of *Glut-3* has been widely established in mice and rats’ brain region such as the hippocampus, cerebral cortex, striatum and cerebellum [31]. Using the *Glut-3* gene as a biomarker for neurodegeneration can be ample to weigh the association in T2DM. Additionally, the *claudin-5a* gene can show the brain integrity localized at the blood–brain barrier (BBB) [32]. The blood–brain is an interface which consists of specific transporters to maintain gradients for organic molecules, metabolites and nutrients across the BBB [33]. The key event of insulin resistance leading to AD appears to be the disintegration of BBB and the loss function of tight junctions due to the disarrangement of glucose homeostasis. There is growing evidence showing similarities of the BBBs between zebrafish and mammals, supporting the model in validating the neurodegenerative study [34,35,36].

To correlate our findings from the insulin resistance zebrafish model, we further investigate with ribonucleic acid (RNA) transcriptomic profiling. The data acquisition upon the validation of the insulin resistance model was broadened by using an algorithm on the database for annotation, visualization and integrated discovery (DAVID)-knowledgebase. The gene ontology (GO) of functional genes and the Kyoto Encyclopedia of Genes and Genomes (KEGG) pathway from the gene set enrichment and differentially expressed analysis were evaluated in this study.

The association of insulin resistance that modulates the glucose homeostasis and other pathophysiological changes that lead to neurodegeneration will be assessed in a zebrafish model. The larval-zebrafish embryogenesis (*Danio rerio*) is an emerging vertebrate model for the in-vivo imaging of biological phenomena at subcellular, cellular and physiological levels [37,38,39].

## 2. Results

### 2.1. Glucose Levels’ Measurement

**Figure 1:**
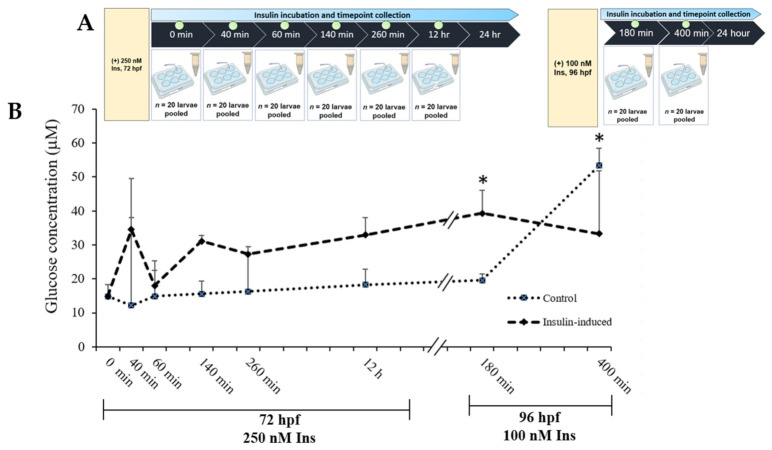
(**A**) Layout of experimental protocol of insulin inductions in zebrafish larvae. Larvae were induced with 250 nM insulin at 72 hpf followed by sampling after completion of the first insulin induction at 0, 40, 60, 260 and 12 h. The second induction with 100 nM insulin at 96 hpf followed by sampling after the second insulin induction was completed at 180 and 400 min. The control group was immersed in E3 medium. (**B**) The effects of post-insulin induction on a glucose dynamic study by zebrafish larvae during the first and second insulin inductions. The x-axis values on the scale are in minutes or hours while the y-axis values on the scale are in micromolars. The data are expressed in (mean ± SEM). Statistically significant values are expressed as * *p* < 0.05.

A time points study was designed to elucidate the dynamic of glucose levels towards the high insulin induction in zebrafish larvae at 72 hpf and 96 hpf. At 0 min, both the control and the insulin-induced group started at 14.85 μM ± 3.48. An increment in glucose levels was seen at 40 min and dropped at 60 min. Additionally, there was an increment upon 24 h of the second induction (100 nM insulin), significantly higher than the control group at 180 min, which exhibited 39.32 μM ± 1.95 and 19.57 μM ± 6.72, respectively. Towards the end of glucose levels’ analysis, the insulin-induced group dropped significantly lower than the control group (Figure 1).

### 2.2. Gene Expression Analysis

**Figure 2:**
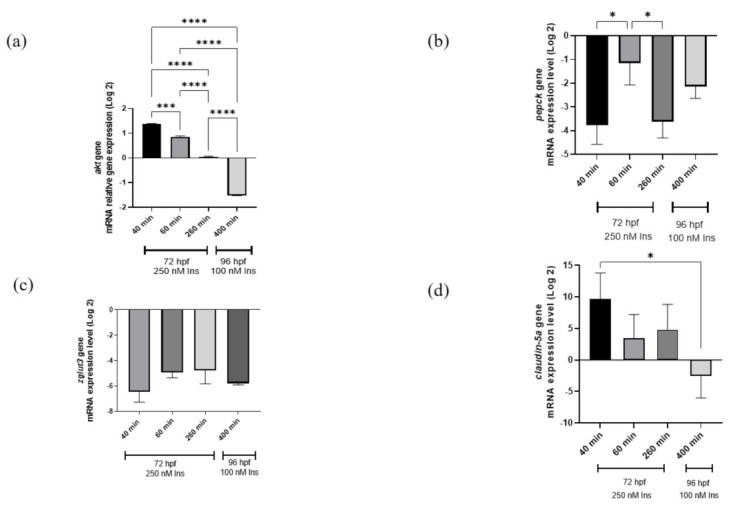
qRT-PCR analysis for insulin resistance model validation with (**a**) *akt*, (**b**) *pepck*, (**c**) *zglut3*, and (**d**) *claudin-5a* genes expression on the effects of high insulin induction for 250 nM at 72 hpf and re-challenging after 24 h with 100 nM insulin at 96 hpf. *Beta-actin*, a housekeeping gene, was used for normalization. Data represent the mean ± SEM; *n* = 3 independent replicates with 20 larvae in each replicate. Statistical significance, as analyzed by One-way ANOVA with Tukey multiple comparison groups test, is expressed as (*) *p* < 0.05, (***) *p* < 0.001 and (****) *p* < 0.0001.

Quantitative PCR analysis was used in this study, showing the time point group designation as shown in the references from previous studies [25,27]. The study is important in terms of glucose dynamic study at different time points to validate that hyperinsulinemia induction can induce hyperglycemia and the transposition of insulin signaling genes (*pepck* and *akt*) affecting two genes involved in neurodegeneration (*zglut3* and *claudin-5a*). The relative *akt* gene mRNA expression levels were significantly upregulated (*p* < 0.001) at 40–60 min and downregulated at 260 min of 250 nM insulin and 400 min of re-challenging with 100 nM insulin (*p* < 0.0001). Meanwhile, the *pepck* gene mRNA expression levels were significantly downregulated (*p* < 0.05) with three consecutive time points but not in the second induction. In contrast to the *claudin-5a* gene, the mRNA expression levels showed significant downregulation 40 min after the first insulin induction and 400 min (*p* < 0.05) after the second insulin induction (Figure 2).

### 2.3. Lipid Distribution Analysis

**Figure 3:**
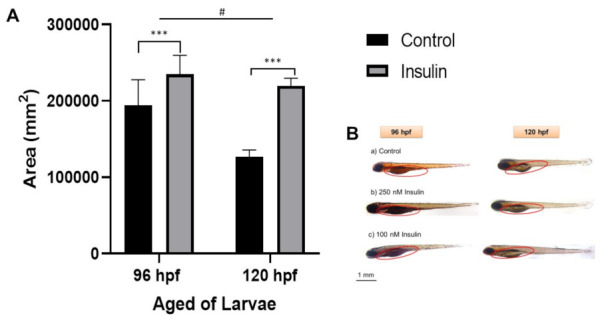
Representative image of whole-mount Oil Red-O (ORO) staining for comparison between control and insulin-induced group larvae (triplicate, pool of three zebrafish per sample). (**A**) The relative area was counted in six replications using ImageJ software for the total area each of area yolk sac to its yolk extension in each larva (mean ± SEM). Two-way ANOVA was applied for the analysis and *p*-value is expressed as (***). *p* < 0.001 is comparing to control and (#) *p* < 0.05 is comparing to the age factor. (**B**) The image of ORO staining in 96 and 120 hpf zebrafish in the lateral view is shown with the enlargement in yolk sac size. The circle is a yolk sac area measurement scale at 1 mm.

The area of the yolk sac was measured to elucidate the phenotype based on the relative lipid distribution after insulin induction in the visualization of a semiquantitative analysis (Figure 3). Lipid distribution was analyzed based on the completion of 96 hpf of the insulin induction at 250 nM insulin immersion and 120 hpf of the second insulin induction at 100 nM insulin-induced immersion. Sample collection was timed at the end of the 24 h cycle of insulin absorption. The first sample post induction with 250 nM insulin at 72 hpf was collected at 96 hpf and stained with Oil Red O (ORO), a neutral lipid stain for whole-tissue staining. Then, the second sample post induction with 100 nM insulin at 96 hpf was collected at 120 hpf and also stained with ORO. The yolk sac and yolk sac extension, along with the forebrain, otic vesicle, and around the eye, was strongly stained with ORO [40], indicative of hyperinsulinemia leading to an accumulation of lipid in the yolk sac and yolk sac extension of the zebrafish larvae. Jun Ka et al., (2020) reported that diabetes predisposes to hyperlipidemia with elevated lipid deposition in vessels and the yolk sac [41]. Our study showed that there was a significant increase in yolk sac area in the insulin-induced group of 235,181 ± 14,105 mm^2^ compared to the control group of 219,941 ± 5544.01 mm^2^. At different age groups of 96 and 120 hpf, there were significant differences in the increase in yolk sac area with the mean ± SEM (41,436 ± 12,512 mm^2^).

### 2.4. Oxidative-Stress Measurement

**Figure 4:**
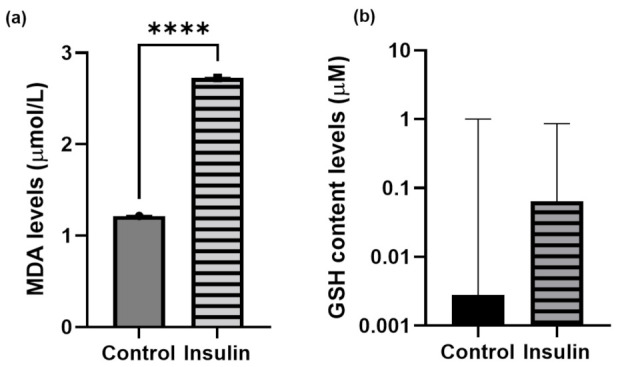
Unpaired *t*-test analysis. Data of (**a**) MDA and (**b**) GSH levels are expressed in mean ± SEM with three independent replications for each group pooled with 20 larvae. Statistical analyses are expressed as *p* < 0.001 (****).

Reactive oxygen or nitrogen species are the precursors of oxidative stress. MDA is a byproduct of the reaction of lipid hydroxides which react against the free radicals during lipid peroxidation [42]. Meanwhile, zebrafish have an antioxidant system such as GSH which is possessed by zebrafish to act as a free radical scavenger [43]. Insulin resistance is thought to enhance and trigger the oxidative stress in zebrafish larvae. Therefore, in this study, MDA and GSH were used as indicators of oxidative stress to confirm the insulin resistance model. It has been suggested that insulin resistance may induce the elevation of (a) MDA and (b) GSH levels. The mean ± SEM difference in MDA content levels in high insulin induction between the control and insulin-induced groups was 1.508 ± 0.0037 μmol/L with a significant difference (*p* < 0.0001) (Figure 4a). However, there was no significant difference between the two groups for the GSH content observed in zebrafish larvae (Figure 4b).

### 2.5. DAVID Functional-Annotation Analysis from RNA-seq

**Figure 5:**
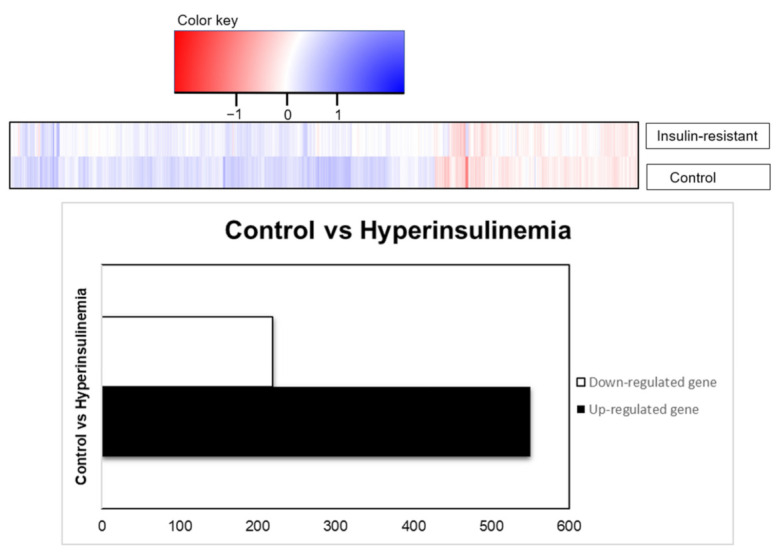
RNA transcriptomic profiling, based on differential expression genes presented as total genes, were downregulated (219 genes) or upregulated (550 genes) based on the adjusted *p*-values (*p* < 0.05).

Differential expression analysis (DEA) with the up- and downregulated genes from the Log-fold change (LogFC) and the adjusted *p*-value are to validate the mechanism changes upon the high insulin inductions. RNA sequencing was performed using the transcription profile. Given 32,521 genes from the expression in zebrafish species, there were 769 genes which were differentially expressed based on the statistical design, adjusted *p* < 0.05, and FDR < 1.0. The result is presented in the table with the gene set enrichment using DAVID with the indicated KEGG-signaling pathways. Out of 769 genes, 219 genes were downregulated, and 550 genes were upregulated by insulin resistance (Figure 5). There were 490 genes upregulated and 167 genes downregulated recognized in the DAVID platform, with *Danio rerio* background implied in this study. Statistical analysis of gene enrichment, based on kappa threshold > 0.03, ranks the biological significance of gene groups based on EASE scores (*p* < 0.05).

To visualize the functional signaling pathways involved in this study, a set of differential expression genes that are significant at *p* < 0.05 were enriched to classify them into top gene ontology (TopGO) (Table 1 and Table 2) and KEGG pathways (Table 3, Table 4, Table 5 and Table 6). The upregulated functional KEGG pathway was MAPK and Wnt/Ca^2+^ signaling, while the downregulated one was metabolic pathways. The insulin-signaling pathways showed up- and downregulation. In addition, from the TopGO term, the functions of the biological process (BP), cellular component (CC) and molecular function (MF) are attributed as significant for the gene set enrichment analysis as shown in Table 1 and Table 2.

## 3. Discussion

In this study, the 72 hpf and 96 hpf larval stages of zebrafish were used to explore the impact of insulin resistance associating with neurodegeneration mechanisms. While previous studies had utilized morpholino-knockdown or transgenesis to induce insulin resistance [25,44,45,46], we used insulin solution by immersing 20–30 zebrafish larvae per group. This method provides a robust and less invasive method as described in a previous study [27]. However, they reported different concentrations of insulin at different times of exposure. On the other hand, the immersion method using paracetamol was established in zebrafish larvae and described by van Wijk et al., (2019), which occurred entirely by oral ingestion and trans-gill absorption at the exposure in 3 and 4 days post fertilization (dpf) [47]. This indicates that zebrafish larvae are able to directly absorb any treatment in the form of a solution for the purpose of the intervention study.

Our present data showed that this insulin resistance model exerted the desired hypoglycemic–hyperglycemic effects; we performed a time-course measurement of glucose levels in the zebrafish larvae. Insulin resistance was induced in zebrafish by immersion in insulin solution at 72 and 96 hpf. After the second induction of 100 nM insulin at 96 hpf, the glucose level in the treated group was significantly increased (*p* < 0.05) 180 min after insulin immersion. However, 400 min after the insulin immersion, the glucose level was significantly decreased compared to the control group (*p* < 0.05). At 3 and 4 dpf, the zebrafish larvae still used endogenous nutrients from the yolk sac for growth and organ development [48]. We postulate that during the initial induction of insulin immersion, the effects of hyperglycemia were not observed due to the availability of endogenous nutrients in the yolk sac of zebrafish larvae, which interfered with exogenous insulin activity. Studies on mammalian metabolic programming have shown that nutrient supply at crucial developmental stages early in life could have genetic and physiological consequences over time [49,50,51,52]. As a result, physiological alterations such as glucose homeostasis and molecular changes based on the relevant genes were evaluated in later transcriptome-profiling studies to confirm the insulin resistance established in the zebrafish model.

To validate the mechanism of insulin resistance via glucose levels’ tolerance to high insulin, we measured the mRNA expression genes at insulin-signaling levels such as *akt* and *pepck* genes. A high dose of insulin may result in reduced beta cell islet and cell sensitivity and promote insulin-resistant individuals. Nam et al., (2021) revealed difficulties in estimating insulin induction time due to the diligence of insulin exposure to endogenous nutrients stored in the yolk sac [27]. It has been reported that endogenous nutrients are fully used in zebrafish larvae at 3 dpf, suggesting that insulin treatment should be extended to 96 hpf to create a T2DM model [27].

It is well documented that exposure of zebrafish to high levels of insulin has successfully induced insulin [25,27,53]. To better characterize how the state of insulin resistance can disrupt insulin-signaling pathways, *akt* and *pepck* were the chosen genes in this study. The *akt* gene, known to be a central component in the signaling pathways activating the kinase family for assessing early stage insulin resistance [54,55], was significantly downregulated over time after insulin immersion.

Meanwhile, *pepck* mRNA expression has been shown to decrease 20–40 min after insulin stimulation [25], and this effect is conserved in zebrafish [25,26,49]. In parallel, our study showed that the significant downregulation of the *pepck* gene occurred at 40–260 min after insulin immersion. *Pepck* is known to be a rate-limiting enzyme in gluconeogenesis and a useful marker for the effectiveness of insulin exposure in the treated larvae [56]. Our study demonstrated *pepck* downregulation at the cellular level, suggesting that insulin signaling is suppressed by high insulin induction. As seen from the glucose dynamic results, increasing glucose levels in zebrafish larvae between 260 min and 12 h after initial insulin induction (250 nM insulin) can result in transient hyperglycemia [25]. Recent data support the findings of significant *pepck* expression downregulation at 260 min but not 400 min. Ghaddar and Diotel (2022) reviewed the implications of the zebrafish model of chronic hyperglycemia that could modulate insulin behavior [57]. Likewise, our zebrafish larval model showed the effects of hyperglycemia. In contrast, the *akt* gene obtained from qPCR was downregulated in the insulin resistance model at 400 min post immersion. The oxidative-stress assays and NGS were found to contradict the outcome from the qPCR. The results from the glucose dynamic study versus NGS did not repress the *akt* gene instead of the *forkhead box protein O1* (*foxo1*) gene in these findings. Talchai and Accili (2015) reported that mice lacking the *foxo1* gene model had reduced insulin sensitivity but continuously increased beta-cell mass in the pancreas [58]. Similar to the previous study conducted by Al-Masri et al., (2010), using a fetal pancreatic tissue knockdown of FOXO1 siRNA demonstrated the enhancement in *akt* phosphorylation in the insulin-signaling mechanism [59]. In addition, transcriptome profile data enriched by the DAVID indicate upregulation of the *foxo1b* gene and downregulation of fructose-1,6-bisphosphatase 2 (*fbp2*) gene. The *foxo1* gene has been established in the mouse model of diabetic cardiomyopathy which upregulates the beta isoform of the myosin heavy chain (β-MHC) but inhibits PI3K pathways, resulting in impaired energy metabolism [60]. We propose that *foxo1b* activation may stimulate the physiological disturbances and this occurrence may cause energy deprivation in the cells. However, *fbp2*, a gluconeogenic enzyme, exhibited the inhibition of gluconeogenesis in cancer cells [61] which, in our findings, shows that this gene is downregulated. It could be explained that the endogenous glucose is used entirely in the post-insulin immersion. Prolonged exposure to insulin is proposed in future studies to postulate the activation of *foxo1*, which might inhibit *fbp2*, and thus aggravate glucose homeostasis.

In addition, the *claudin-5a* gene was chosen to ascertain the association of insulin resistance and neuropathology that are involved in the BBB. In zebrafish, the *claudin-5a* gene is present in the choroid plexus which resembles the BBB structure [62]. BBB tight junctions are the basic framework to block unwanted traits between blood and brain [32]. *Claudin*, *occludin* and junctional adhesion molecules are assembled as integral proteins at the transmembrane of the BBB [63]. The *claudin-5a* gene was chosen in the current study because it has orthologues in the zebrafish genus [34]. *Claudin-5a* was found in the ventricular zone of the midbrain, hindbrain, and epiphysis as well as in the blood arteries of the brain as early as 1–2 dpf [32]. The drastic downregulation of the *claudin-5a* gene observed in our study suggests that the gene was inhibited by insulin resistance, implying the second post-insulin immersion that caused further BBB disintegration.

The ORO-staining approach was also used to study lipid distribution in the yolk sac by observing the oil red droplets accumulated in the yolk sac of zebrafish. Insulin resistance can lead to lipogenesis, as shown by the rapid growth of zebrafish in the first and second insulin inductions. Evidently, the yolk sac of zebrafish larvae induced with insulin resistance was significantly enlarged. It is necessary to transfer endogenous nutrients from the yolk sac to the embryonic larvae stage, but they degrade as the tissue grows [64]. This finding showed how lipogenesis, which is the conversion of fatty acids and glycerol into fats, evolved as a result of the insulin-resistant conditions disrupting glucose homeostasis. Rocha et al., (2014) reported high glucose exposure caused the lipogenesis enzyme to increase at 4–6 dpf zebrafish larvae [26]. The phenomenon is clearly stated by Flannery et al., (2012), which is hepatic de novo lipogenesis promoted by insulin resistance that occurs in skeletal muscle [65].

Interestingly, the gene ontology involved in lipid metabolism is downregulated in insulin resistance. It can be explained by a variety of metabolic pathways involving leptin signaling, gluconeogenesis and lipid metabolism [25,66]. Genes involved in insulin signaling were found to be upregulated (*foxo1b*, *bach1a*, *mafgb*, *pou6f2*, *mafk*, *fosl1a*, *sp8b*, *hsf4*, *crebrf*, *gatad2b*, *lrrfip1b*, *her4.2* and *rxrab*) and a few were downregulated (*ehf* and *dmrt1*) as presented in differential expression enrichment. Based on transcriptomic profiles with the selected signaling pathways active in the insulin resistance condition, we predicted that the central nervous system (CNS) might be associated with metabolic disarray. It could be postulated that the CNS might have a strong association with metabolic disorders based on transcriptome profiles with selected signaling pathways affected by the insulin resistance.

In addition, insulin resistance promotes cell starvation, and persistence of insulin resistance can lead to an increment in free radicals. It is known that free radicals distress metabolic regulation and signaling processes. The hallmark of cellular damage from free radicals is the breakdown of deoxyribonucleic acid (DNA), proteins and lipid membranes [67]. MDA, a biomarker of oxidative stress, can be produced by lipid peroxidation in reactive oxygen species (ROS). According to the current findings, the MDA activity was substantially higher in the experimental group than in the control group. ROS damage has also been linked to a mitogen-activated protein kinase (MAPK) pathways, erb-b2 receptor tyrosine kinase-4b and protein kinase C processes, according to gene ontology. A comparable zebrafish model produced with high levels of insulin demonstrated MAPK pathways associated with T2DM [27,68]. The upregulation of MAPK genes and downregulation of the *claudin-5a* gene characterized the association of MAPK signaling in T2DM and neurodegeneration observed in the current study. In addition, the upregulation of amyloid beta (Aβ4) precursor protein-binding-5 family member 3 (*appb3*) and tau tubulin kinase 1 (*ttbk1*) was also observed in this study. The breakdown of the epithelium of the choroid plexus caused the senile plaque that is the consequence of the accumulation of amyloid beta. The abnormal insulin level is believed to enhance the upregulation of *appb3* and *ttbk1*, which causes the choroid plexus to be disintegrated, leading to neurodegeneration [35,69]. Unfortunately, GSH levels could not be observed significantly in this study, although the trend of the hyperinsulinemia group was increased compared to the control. We were able to understand that GSH protects against oxidative damage which is acting as a scavenger of free radical attack [42,70]. Since insulin resistance leads to ROS and mitochondrial dysfunction, the detoxification response to the biological process of GSH appears to be higher in the treated group. Nevertheless, the GSH levels, which in this study are attributed insignificantly to the insulin resistance due to the dose–response effect of the time exposure of insulin, are not sufficient.

Wnt/Ca^2+^ signaling is known to be an evolutionarily conserved pathway in embryonic and adult life that is correlated to the critical pathway for cognitive function [71]. Our recent data have shown that high insulin inductions can upregulate the Wnt/Ca^2+^ pathway via the wnt5a pathway and the *prkcg*-gene-activated nuclear factor of activated T cells (NFAT). Wnt protein-encapsulated *wnt5a* can modulate inflammation via protein kinase. An elevation in NFAT results in mild cognitive impairment [72]. Zaulkffali et al., (2019) also showed that insulin resistance in neuronal cells activates GSK3beta-mediated tau hyperphosphorylation, which is a biomarker of Alzheimer’s disease (AD). It is evident that insulin-resistance-impaired Wnt/Ca^2+^ signaling leads to GSK3beta activation and the upregulation of *appb3* and *ttbk1*, which initiates neurofibrillary tangle formation. Despite the in vitro and in vivo models validating the cellular levels of this mechanism, extensive studies of human Wnt/beta-catenin signaling from the brain tissues of AD patients have been performed [71,73]. The *Dikkopf-1* (*Dkk1*) inhibitor gene for Wnt/Ca^2+^ was shown to be upregulated in degenerating neurons derived from AD brain tissue, suggesting that Wnt/beta-catenin played a significant role in insulin resistance [71,74].

Insulin resistance was found to be a precursor to neurodegeneration in T2DM, which can be mimicked in zebrafish larvae induced with high insulin. At the cellular mRNA level, interactions of the differential expression genes, phenotypic function assays and oxidative-stress measurements of the architecture of T2DM provide insight into the potential pathways of neurodegeneration. Our results suggest that MAPK signaling and Wnt/Ca^2+^ are the central pathways in insulin resistance incorporated with oxidative stress, leading to the upregulation of neurodegeneration-associated *appb3* and *ttbk* genes.

## 4. Materials and Methods

### 4.1. Reagents and Equipment

We acquired Insulin Human Recombinant (BioVision, San Francisco, CA, USA), ORO dye (Catalogue number: O0625, Sigma-Aldrich, St-Louis, MO, USA), PRImeZOL^™^ reagent (Catalogue number: AN1102, Canvax, Cordoba, Spain), isopropanol, absolute ethanol for molecular biology grade (Merck, Darmstadt, Germany), and phosphate-buffered saline (PBS). For microscopic observation and analysis, a semi-automated Leica M205A Microsystem (Heerbrugg, Switzerland) with integrated Leica Application Suite (LAS) 4.6 software (Heerbrugg, Switzerland) was used.

### 4.2. Zebrafish Husbandry

Fish were kept and housed in the Zebrafish Laboratory under a 14 h light and 10 h dark photoperiod cycle in a recirculating aquaculture system (Techniplast ZebTEC, Buguggiate (VA), Italy). Water factors such as temperature, pH and conductivity were monitored daily. The fish were fed with dry food pellets and live *Artemia salina* three times a day. Breeding was established the day before egg collection with a 2:1 ratio of adult males to females. After successful spawning the next day, the embryos were collected in the Petri dish with system water containing 0.1% methylene blue and incubated at 28 °C.

### 4.3. Insulin Induction

**Figure 6:**
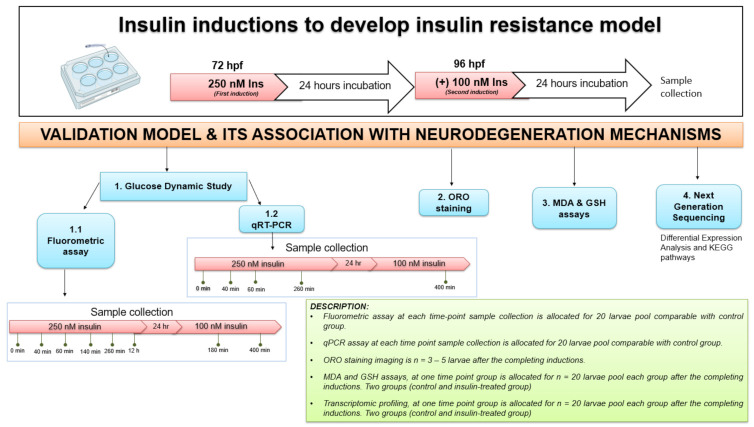
The experimental design for insulin resistance zebrafish larvae model at 72 and 96 hpf with 250 nM and 100 nM insulin induction, respectively, by immersion technique. The groups at each time point were assessed in triplicate (*n* = 3). The variation in sampling timing after insulin immersion is revealed. Dynamic glucose assay samples for post-insulin immersion were collected at 0 min, 40 min, 60 min, 140 min, 260 min and 12 h at first induction of 250 nM insulin. After the second insulin induction of 100 nM insulin, samples were taken at 180 and 400 min. Quantitative RT-PCR (qRT-PCR) analysis samples were collected at 40, 60 and 260 min of 250 nM insulin induction and at 400 min of 100 nM insulin induction. After each insulin induction, the ORO staining was scored at 96 hpf and 120 hpf after the first and second insulin induction. MDA, GSH and next-generation sequencing (NGS) for transcriptome profiling were developed to connect to the mechanisms of neurodegeneration. The control used was E3 medium containing zebrafish buffer. *hour-post-fertilization (hpf)*; *Insulin (Ins)*.

Healthy zebrafish embryos were transferred to 6-well plates, each well containing 20 embryos/group. The zebrafish larvae were induced at 72 and 96 h post fertilization (hpf) with 250 nM and 100 nM insulin (human recombinant, BioVision, San Franscisco, CA, USA), respectively, in a total volume of 1 mL per well, based on the concentration validated in Marin-Juez et al., (2014) [25] or a control group in E3 medium (5 mM NaCl, 0.17 mM KCl, 0.33 mM CaCl_2_ and 0.33 mM MgSO_4_). All solutions were refreshed daily to every 24 h cycle at room temperature. The main parts of a typical construction are shown in Figure 1 with the description provided. First, the insulin resistance development model in zebrafish larvae was established using three parameters (fluorometric assay, qPCR analysis and ORO staining) at 72 and 96 hpf after insulin immersion with time point groups. Second, after validation of the insulin resistance model, next-generation sequencing (NGS) of transcriptome profiling analysis was measured following functional assays such as MDA and GSH assays (Figure 6).

### 4.4. Glucose Dynamic Study

Glucose levels in zebrafish larvae were measured based on eight time-dependencies shown in Figure 1, with each group of time points containing 20 larvae from control or insulin-inducing groups. A fluorescence-based enzymatic detection kit was used to measure the glucose levels in zebrafish larvae (Catalogue number: STA-681, Cell Biolabs, Inc., San Diego, CA, USA).

### 4.5. Quantitative PCR (qPCR) Analysis for the Relative Genes of Interest Expression Analysis

Twenty control and insulin-induction timepoint groups of zebrafish larval lysates were homogenized, and total RNA was extracted using PRImeZOL^TM^ reagent (Catalogue number: AN1102, Canvax, Cordoba, Spain). Purity and integrity of total RNA were assessed using NanoDrop 2000 (Thermo Fisher Scientific, Waltham, MA, USA) and gel electrophoresis to confirm the integrity of 28S and 18S RNA.

A two-step quantitative RT-PCR was adopted to quantify the relative expression of the GOIs. cDNA (PCRBIOsystem, London, UK) was synthesized and preferred for sequencing the verification primers on the NCBI website or from the previous publications (Appendix A). The expression levels of the insulin-signaling markers were selected using *akt*, *pepck* and *zglut3* and BBB integrity markers (*claudin-5a*). The cDNA templates were used in qPCR amplification reactions with gene-specific primer pairs. PCR amplifications were performed in 20 μL reactions containing cDNA prepared from an RNA template based on 10-fold dilution factor and 200 nM each of the gene-specific forward and reverse primer, and 10 μL of qMAXSen™ Green qPCR Mastermix (w/out ROX™) (Catalogue number: E0354, Canvax, Cordoba, Spain) was generated. Amplified signals from duplicate reactions were detected and analyzed using the MiniOpticon BioRad analyzer (California, USA) and CFX manager software (California, USA). The amplification protocol used was as follows: initially, withhold phase at 95 °C for 5 min denaturation and enzyme activation, perform 40 cycles of 95 °C for 3 s and 61 °C for 30 s, and set the melting curve phase to 65 °C for 60 s and 95 °C for 15 s. The housekeeping gene for all primers was beta-actin, with qPCR data normalized to the beta-actin levels using the Livak method with the ΔΔCt value [75]. Three independent experiments were performed, and the quantitative data obtained were averaged based on the quantification cycle (Cq) values, which were used to calculate the relative expression gene.

### 4.6. Oil Red O (ORO) Staining

The working solution of Oil Red O (ORO) reagent was prepared at 0.3% of ORO powdered form (O0625, Sigma-Aldrich, MO, USA) in 60% isopropanol filtered using Whatmann #1 which is stable for 2 h. Larvae were fixed in 4% of paraformaldehyde in phosphate-buffered saline (PBS) overnight at 4 °C before incubation in 60% of isopropanol. The larvae were washed three times with PBS. Then, they were pre-incubated in 60% of isopropanol for 30 min and stained with freshly filtered 0.3% ORO reagent for 3 h. The semi-automated Leica M205A Microsystem with integrated LAS 4.6 software was used for microscopic observation and image acquisition. The yolk sac and yolk sac extension area of the larvae were measured using ImageJ software (National Institutes of Health, USA).

### 4.7. Malondialdehyde (MDA) Assay

Malondialdehyde (MDA) activity was measured by the level of lipid peroxidation and exhibits cellular damage indirectly by thiobarbituric acid (TBA). The protocol was based on the manufacturer’s Biochemical Assay Kit (Catalogue number: E-BC-K025-M, Elabscience® Biotechnology Inc, Houston, Texas, USA). We measured the MDA contents in zebrafish whole embryos involved in the reaction between MDA and TBA to form the red complex, that could be detected by a UV-VIS spectrophotometer at 532 nm.

### 4.8. Glutathione (GSH) Assay

Glutathione (GSH) levels were assayed using the method described by the manufacturer. The protein concentration measured by the Bradford method was used to correct the activity of antioxidant enzymes (Catalogue number: STA-312, OxiSelect™ Total Glutathione (GSSG/GSH) Assay Kit, Cell Biolabs, Inc., San Diego, CA, USA). The measurement of GSSG/GSH using a kinetic assay with optical density (OD) at 405 nm.

### 4.9. RNA-Seq Transcriptomic Profilings

Twenty larvae per group from the insulin resistance model as described in (Figure 6) were homogenized in 500 μL of PRImeZOL^TM^ reagent (Canvax, Cordoba, Spain), and total RNA was extracted according to the manufacturer’s instructions. Total RNA per group was quantified and qualified using the Agilent 2100 Bioanalyzer (Agilent Technologies, Palo Alto, CA, USA) and the Nanodrop of RNA purity detection (Thermo Fischer Scientific, Inc.), and 1% agarose gel was validated. One μg of total RNA with an RNA integrity number (RIN) value greater than 6.5 was used for the following library preparations, which were made according to the manufacturer’s protocol. Poly(A)-mRNA isolation was performed using the Poly(A) mRNA Magnetic Isolation Module or rRNA removal kit. The mRNA fragmentation and priming were performed using First-Strand Synthesis Reaction Buffer and Random Primers that provided by the GENEWIZ (Azenta Life Science, Guangzhou, China). First-strand cDNA was synthesized using ProtoScript II Reverse Transcriptase and the second-strand cDNA was synthesized using Second Strand Synthesize Enzyme Mix. The bead-purified double-strand cDNA was then treated with End Prep Enzyme Mix to repair both ends and add a dA-tailing in one reaction, followed by a T-A ligation to add adaptors on both ends. Size selection of adaptor-ligated DNA was then performed using beads, and fragments of approximately 420 base pairs (the insertion size of 300 base pairs approximately) were recovered. Each sample was then amplified by PCR for 13 cycles using P5 and P7 primers, with both primers carrying sequences capable of hybridizing to flow cells to perform bridge PCR, and the P7 primer carried a six-base index which allowed multiplexing. The PCR products were purified using beads, validated using a Qsep100 (BiOptic, Taiwan, China), and quantified with a Qubit3.0 Fluorometer (Invitrogen, Carlsbad, CA, USA). Then, libraries with different indices were multiplexed and loaded onto an Illumina HiSeq instrument according to the manufacturer’s instructions (Illumina, San Diego, CA, USA). Sequencing was performed using a 2 × 150 bp paired-end (PE) configuration; image analysis and base calling were conducted by the HiSeq Control Software (HCS) + OLB + GAPipelines-1.6 (Illumina) on the HiSeq instrument. The sequences were processed in Fastq file and pre-analyzed by GENEWIZ (Azenta Life Sciences, Guangzhou, China). The set of genes was analyzed based on differential expression analysis with LogFC and the Benjamini and Hochberg (1995) algorithm [76]. *p*-values < 0.05 were differentially expressed between groups (DESeq2 Bioconductor package). The raw RNA-seq data were deposited with NCBI BioProject ID under the accession number PRJNA743929.

### 4.10. Functional and Pathway Enrichment Analysis

GOSeq (v1.34.1) [77] was used to identify gene ontology (GO) terms that annotate a list of enriched genes with a significant *p*-value < 0.05. The list of enriched genes was submitted to the Database for Annotation, Visualization and Integrated Discovery (DAVID) database (https://david.ncifcrf.gov/tools.jsp, accessed on 8 December 2021) to generate the gene functional classification covering biological process (BP), cellular component (CC) and molecular function (MF) domains in Top Gene Ontology (TopGO). The functional classification and pathways from the Kyoto Encyclopedia of Genes and Genomes (KEGG) provide a rapid means to organize large lists of genes into functionality related to T2DM disease associated with neurodegeneration to help unravel the biological content captured by stringency level integrated with DAVID. In this study, medium stringency generates balanced results of functional groups of genes with kappa values (ranging from 0 which is considered weak to 1 which is considered strong) to identify a meaningful biological value. The threshold of the EASE score, a modified Fisher exact *p*-value, was used for gene enrichment analysis. Fisher exact *p*-value = 0 represents perfect enrichment.

### 4.11. Statistical Analysis

Statistical analyses were performed using a GraphPad Prism (version 9, San Diego, CA, USA). Data were expressed as the mean ± standard error of the mean (SEM). Differential statistical significance was determined using Dunnett’s test, one-way ANOVA with Tukey’s multiple comparison groups and unpaired *t*-test analysis in each of the parameters. The level of statistical significance was set at a *p*-value < 0.05.

## 5. Conclusions

In this study, we used a larval-zebrafish model of insulin resistance to ensure that it precisely reflects T2DM pathogenesis and may be associated with neurodegenerative mechanisms. Our insulin resistance model has numerous advantages, including (1) rapid development of T2DM symptoms, (2) significant genetic homology with humans, (3) pathological evidence in genetic analysis on the human platform. Our findings imply that the insulin resistance model in T2DM orchestrated by MAPK/Wnt-Ca^2+^ pathways could be a useful animal model for phenotype-driven drug discovery against diabetes, as well as a targeted treatment modality to unravel the mechanism associated with neurodegeneration.

## Figures and Tables

**Table 1:** Top 10 upregulated gene sets enriched based on gene ontology (GO) term with adjusted *p*-value EASE score.

Upregulated			
TopGO	Term	Total Genes	Adjusted *p*-Value EASE (<0.05)
GO_BP	Intracellular signal transduction	48	2.50 × 10^−10^
	Calcium ion import	6	9.50 × 10^−5^
	Regulation of ion transmembrane transport	12	1.10 × 10^−4^
	Homophilic cell adhesion via plasma membrane adhesion molecules	14	2.40 × 10^−4^
	Calcium ion transmembrane transport	9	4.40 × 10^−4^
	Calcium ion transport	8	4.40 × 10^−4^
	Transmembrane transport	21	3.90 × 10^−3^
	Axonogenesis involved in innervation	3	6.10 × 10^−3^
	Regulation of endocytosis	4	6.50 × 10^−3^
	Positive regulation of kinase activity	6	1.20 × 10^−2^
GO_CC	Plasma membrane	74	2.00 × 10^−6^
	Voltage-gated calcium channel complex	8	7.90 × 10^−6^
	Integral component of plasma membrane	39	6.30 × 10^−5^
	Membrane	139	3.80 × 10^−4^
	Basal plasma membrane	4	9.10 × 10^−4^
	Axon	10	1.00 × 10^−3^
	Axonal growth cone	3	7.80 × 10^−3^
	Integral component of plasma membrane	128	8.50 × 10^−3^
	Receptor complex	8	1.20 × 10^−2^
	Synaptic vesicle	6	1.20 × 10^−2^
	Synapse	15	1.60 × 10^−2^
GO_MF	Voltage-gated ion channel activity	12	2.90 × 10^−5^
	Voltage-gated calcium channel activity	8	4.10 × 10^−5^
	Tau protein binding	4	4.30 × 10^−5^
	Metal ion binding	81	6.80 × 10^−5^
	High-voltage-gated calcium channel activity	5	1.10 × 10^−4^
	Ion channel activity	12	1.30 × 10^−3^
	Calcium channel activity	7	3.60 × 10^−3^
	Transmembrane-receptor-protein tyrosine kinase activity	7	1.50 × 10^−2^
	Protein tyrosine kinase activity	7	1.60 × 10^−2^
	Protein kinase activity	18	9.80 × 10^−2^

**Table 2:** Top 10 downregulated gene sets enriched based on gene ontology (GO) term with adjusted *p*-value EASE score.

Downregulated			
TopGO	Term	Total Genes	Adjusted *p*-Value EASE (<0.05)
GO_BP	piRNA metabolic process	6	3.70 × 10^−8^
	Proteolysis	16	2.70 × 10^−6^
	Gene silencing by RNA	4	2.00 × 10^−3^
	Transmembrane transport	11	4.00 × 10^−3^
	Lipid metabolic process	7	1.70 × 10^−2^
	Neutrophil chemotaxis	4	1.70 × 10^−2^
	High-density-lipoprotein-particle assembly	2	2.50 × 10^−2^
	Reverse cholesterol transport	2	2.50 × 10^−2^
	Very-low-density-lipoprotein-particle modelling	2	2.50 × 10^−2^
	Cholesterol efflux	2	8.40 × 10^−2^
GO_CC	Extracellular region	24	6.00 × 10^−8^
	Extracellular space	23	1.50 × 10^−6^
	P granule	5	1.30 × 10^−5^
	pi-body	3	2.00 × 10^−4^
	High-density-lipoprotein particle	3	1.50 × 10^−3^
	Chylomicron	2	2.90 × 10^−2^
	Integral component of postsynaptic specialization membrane	2	5.70 × 10^−2^
GO_MF	Hydrolase activity	30	4.70 × 10^−9^
	Peptidase activity	16	7.3–8
	Serine-type peptidase activity	9	1.30 × 10^−6^
	piRNA binding	3	1.10 × 10^−4^
	Carboxypeptidase	4	3.60 × 10^−4^
	Endoribonuclease activity, producing 5′-Phosphomonoesters	3	3.70 × 10^−4^
	Metallocarboxypeptidase activity	4	4.10 × 10^−4^
	Transmembrane transporter activity	7	7.10 × 10^−3^
	Cholinesterase activity	2	3.00 × 10^−3^
	Sterol esterase	2	4.20 × 10^−2^

**Table 3:** Functional annotation chart based on gene set enrichment analysis with EASE score, a modified Fisher exact *p*-value (*p* < 0.1).

KEGG Pathways from the DAVID Platform	
Upregulated	Downregulated
MAPK-signaling pathway; Calcium-signaling pathway; Vascular smooth muscle contracting; Adrenergic-signaling pathway; GnRH-signaling pathway; Purine metabolism; Neuroactive ligand–receptor interaction.	Metabolic pathways; Starch and sucrose metabolism; D-amino acid metabolism; PPAR-signaling pathway.

**Table 4:**
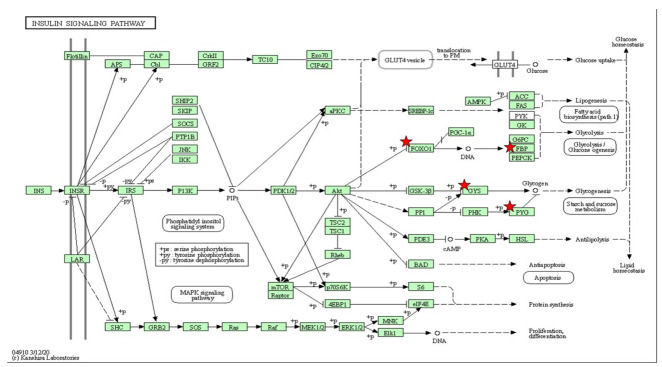
The gene set enriched in insulin signaling and glucose metabolism in the DAVID platform. Insulin-signaling pathway associated with metabolic disorders depicted based on KEGG pathway with the significant differential expressions set enriched (*p* < 0.05). The star (*) indicates the genes involved in the insulin-signaling pathways integrated with the Table 4.

Insulin-Signaling Pathway		
Gene List	LogFC	(*p* < 0.05)
*foxo1b*	2.068	0.05
*bachla*	1.459	0.05
*ehf*	−2.393	0.01
*Magfb*	2.505	<0.01
*pou6f2*	1.812	0.03
*mafk*	1.812	0.03
*fosl1a*	1.404	0.04
*dmrt1*	−5.803	0.03
*sp8b*	2.384	0.04
*hsf4*	1.911	0.01
*crebrf*	3.031	<0.01
*gatad2b*	1.643	0.02
*lrrfip1b*	1.459	0.04
*her4.2*	3.324	<0.01
*rxarb*	7.163	<0.01
**Metabolic pathways**		
*cel.1*	−2.303	<0.01
*cel.2*	−2.464	<0.01
*amy2a*	−1.901	<0.01
*fbp2*	−2.507	<0.01
*chia.6*	−1.372	0.05
*glsl*	−2.033	0.02
*gsta.2*	−1.928	0.01
*gys2*	−1.459	0.03
*hmox1a*	−1.459	0.03
*hao1*	−1.310	0.05
*mthfd1l*	−2.463	<0.01
*mthfd1a*	−1.804	0.01
*pla2g1b*	−1.610	0.02
*paics*	−2.239	<0.01
*pygl*	−1.412	0.03
*sprb*	−2.422	0.02
*si:ch211-264e16.2*	−6.904	<0.01
*si:dkey-266f7.9*	−2.070	<0.01

Gene abbreviations: *forkhead box O1 b (foxo1b), BTB and CNC homology 1%2C basic leucine zipper transcription factor 1 a (bach1a), ets homologous factor (ehb), v-maf avian musculoaponeurotic fibrosarcoma oncogene homolog Gb (mafgb), POU class 6 homeobox 2 (pou6f2), v-maf avian musculoaponeurotic fibrosarcoma oncogene homolog K (mafk), FOS-like antigen 1a, doublesex and mab-3 related transcription factor 1 (fosl1a), sp8 transcription factor b (sp8b), heat shock transcription factor 4 (hsf4), creb3 regulatory factor (crebrf), GATA zinc finger domain containing 2B (gatad2b), leucine rich repeat (in FLII) interacting protein 1b (lrrfip1b), hairy-related 4%2C tandem duplicate 2 (her4.2), retinoid x receptor%2C alpha b (rxrab)- carboxyl ester lipase%2C tandem duplicate 1 (cel.1), carboxyl ester lipase%2C tandem duplicate 2 (cel.2), amylase%2C alpha 2A (pancreatic) (amy2a), fructose-1%2C6-bisphosphatase 2 (fbp2), chitinase%2C acidic.6 (chia.6), glutaminase-like (glsl), glutathione S-transferase%2C alpha tandem duplicate 2 (gsta.2), glycogen synthase 2 (gys2), heme oxygenase 1a (hmox1a), hydroxyacid oxidase (glycolate oxidase) 1 (hao1), methylenetetrahydrofolate dehydrogenase (NADP+ dependent)-1-like (mthfd1l), methylenetetrahydrofolate dehydrogenase (NADP+ dependent) 1a%2C methenyltetrahydrofolate cyclohydrolase%2C formyltetrahydrofolate synthetase (mthfd1a), phospholipase A2%2C group IB (pancreas) (pla2g1b), phosphoribosylaminoimidazole carboxylase%2C phosphoribosylaminoimidazole succinocarboxamide synthetase (paics), phosphorylase%2C glycogen%2C liver (pygl), sepiapterin reductase b (sprb), (si:ch211-264e16.2 (si:ch211-264e16.2), si:dkey-266f7.9 (si:dkey-266f7.9)*.

**Table 5:**
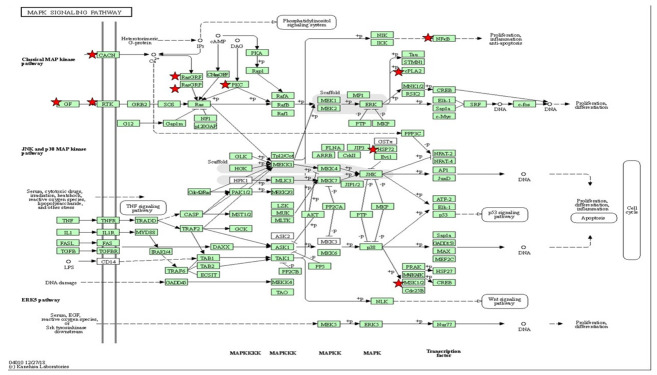
The gene set enriched in the MAPK-signaling pathway in the DAVID platform. MAPK-signaling pathway depicted based on KEGG pathway with the significant differential expressions set enriched (*p* < 0.05). The star (*) indicates the gene involved in the classical MAP kinase pathway integrated with Table 5.

MAPK-Signaling Pathway		
Gene List	LogFC	*p* < 0.05
*rasgrp3*	1.75	0.03
*rasgrf2b*	1.71	0.03
*angpt2b*	2.28	0.05
*erbb2*	1.53	0.04
*erbb4a*	1.63	0.04
*erbb4b*	2.28	0.01
*ntrk2b*	2.72	0.00
*nfkb1*	2.76	0.01
*prkcg*	7.92	0.00
*rps6ka5*	2.38	0.01

Gene abbreviations: *RAS guanyl releasing protein 3 (calcium and DAG-regulated) (Rasgrp3),: Ras protein-specific guanine nucleotide-releasing factor 2b (rasgrf2b);: angiopoietin 2b (angpt2b): erb-b2 receptor tyrosine kinase 2 (erbb2), erb-b2 receptor tyrosine kinase 4a (erbb4a), erb-b2 receptor tyrosine kinase 4b (erbb4b), neurotrophic tyrosine kinase%2C receptor%2C type 2b (ntrk2b), nuclear factor of kappa light polypeptide gene enhancer in B-cells 1 (nfkb1), protein kinase C%2C gamma (prkcg), ribosomal protein S6 kinase%2C polypeptide 5 (rps6ka5)*.

**Table 6:**
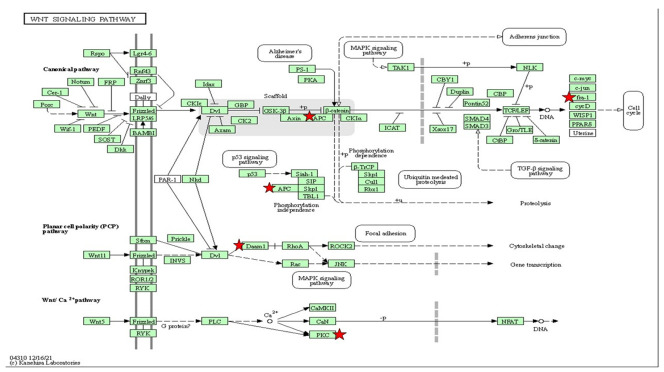
The gene set enriched in the Wnt/Ca^2+^-signaling pathway in the DAVID platform. Wnt/Ca^2+^-signaling pathway depicted based on KEGG pathway with the significant differential expression gene enriched (*p* < 0.05). The star (*) indicates the gene involved in the Wnt/Ca^2+^ pathway integrated with Table 6.

Wnt/Ca^2+^ Pathway		
Gene List	LogFC	(*p* < 0.05)
*Prkcg*	7.92	<0.05
*rps6ka5*	2.38	0.01
*cdc42bpaa*	1.57	0.05
*Camkla*	1.78	0.02
*cdk6*	1.58	0.05
*erbb4a*	1.63	0.04
*Itk*	2.97	0.04
*acvr2ab*	1.96	0.01
*erbb4b*	2.28	0.01

Gene abbreviations: *protein kinase C%2C gamma (prkcg), ribosomal protein S6 kinase%2C polypeptide 5 (rps6ka5), (cdc42bpaa) CDC42 binding protein kinase alpha (DMPK-like) a, calcium/calmodulin-dependent protein kinase 1a (camk1a), cyclin-dependent kinase 6 (cdk6), erb-b2 receptor tyrosine kinase 4a (erbb4a), IL2 inducible T cell kinase (itk), activin A receptor type 2Ab (acvr2ab), erb-b2 receptor tyrosine kinase 4b (erbb4b)*.

## Data Availability

Not applicable.

## Appendix A

**Table A1:** Gene of Interest primers.

Gene of Interest	Primer Sequences	Accession Number
*beta-actin*	3′-CAACGGAAACGCTCATTGC-5′ 5′-CGAGCAGGAGATGGGAACC-3′	Keegan et al., (2002) [78]
*pepck*	3′-ATCACGCATCGCTAAAGAGG-5′ 5′-CCGCTGCGAAATACTTCTTC-3′	NM_214751.1
*claudin-5a*	3′-ATCTTCGTGCTTGTGCCACT-5′ 5′-CAGAGTATGCTTCCCCCGAG-3′	NM_213274.1
*zglut3*	3′-TCGTCAATGTCTTGGCTCTG-5′ 5′-CAACATACATTGGCGTGAGG-3′	ENSDART00000016197
*akt1*	3′-TCG GCA GGTG TCTTC TCAAT-5′ 5′-ACCC ATT GCCATACCACGAG-3′	Sasore and Kennedy (2014) [79]

## References

[1] Cho N.H., Shaw J.E., Karuranga S., Huang Y., da Rocha Fernandes J.D., Ohlrogge A.W., Malanda B. (2018). IDF Diabetes Atlas: Global estimates of diabetes prevalence for 2017 and projections for 2045. Diabetes Res. Clin. Pract..

[2] Ganasegeran K., Hor C.P., Jamil M.F.A., Loh H.C., Noor J.M., Hamid N.A., Suppiah P.D., Manaf M.R.A., Ch’Ng A.S.H., Looi I. (2020). A systematic review of the economic burden of type 2 diabetes in Malaysia. Int. J. Environ. Res. Public Health.

[3] Haile B., Neme K., Belachew T. (2017). Evolution of human diet and effect of globalization on regional diet with emphasis to the Mediterranean diet. Nutr. Food Sci..

[4] Ormazabal V., Nair S., Elfeky O., Aguayo C., Salomon C., Zuñiga F.A. (2018). Association between insulin resistance and the development of cardiovascular disease. Cardiovasc Diabetol..

[5] Shpakov A.O., Derkach K.V., Berstein L.M. (2015). Brain signaling systems in the Type 2 diabetes and metabolic syndrome: Promising target to treat and prevent these diseases. Future Sci. OA.

[6] Matsuzaki T., Sasaki K., Tanizaki Y., Hata J., Fujimi K., Matsui Y., Sekita A., Suzuki S.O., Kanba S., Kiyohara Y. (2010). Insulin resistance is associated with the pathology of Alzheimer disease: The Hisayama study. Neurology.

[7] Suzuki R., Lee K., Jing E., Biddinger S.B., McDonald J.G., Montine T.J., Craft S., Kahn C.R. (2010). Diabetes and insulin in regulation of brain cholesterol metabolism. Cell Metab..

[8] Stanciu G.D., Ababei D.C., Bild V., Bild W., Paduraru L., Gutu M.M., Tamba B.-I. (2020). Renal contributions in the pathophysiology and neuropathological substrates shared by chronic kidney disease and alzheimer’s disease. Brain Sci..

[9] Kim B., Sullivan K.A., Backus C., Feldman E.L. (2011). Cortical neurons develop insulin resistance and blunted Akt signaling: A potential mechanism contributing to enhanced ischemic injury in diabetes. Antioxid. Redox Signal..

[10] Zaulkffali A.S., Razip N.N., Alwi S.S.S., Jalil A.A., Mutalib M.S.A., Gopalsamy B., Chang S.K., Zainal Z., Ibrahim N.N., Zakaria Z.A. (2019). Vitamins D and E stimulate the PI3K-AKT signalling pathway in insulin-resistant SK-N-SH neuronal cells. Nutrients.

[11] Gamba P., Staurenghi E., Testa G., Giannelli S., Sottero B., Leonarduzzi G. (2019). A crosstalk between brain cholesterol oxidation and glucose metabolism in Alzheimer’s disease. Front. Neurosci..

[12] Bing L., Wu J., Zhang J., Chen Y., Hong Z., Zu H. (2015). DHT Inhibits the Aβ25–35-Induced Apoptosis by Regulation of Seladin-1, Survivin, XIAP, bax, and bcl-xl Expression Through a Rapid PI3-K/Akt Signaling in C6 Glial Cell Lines. Neurochem. Res..

[13] Chen Shang Y., Zhong Chong Z., Wang S., Maiese K. (2013). Tuberous Sclerosis Protein 2 (TSC2) Modulates CCN4 Cytoprotection During Apoptotic Amyloid Toxicity in Microglia. Curr. Neurovasc. Res..

[14] Shang Y.C., Chong Z.Z., Wang S., Maiese K. (2012). Prevention of β-amyloid degeneration of microglia by erythropoietin depends on Wnt1, the PI 3-K/mTOR pathway, Bad, and Bcl-xL. Aging (Albany NY).

[15] Tang Z., Bereczki E., Zhang H., Wang S., Li C., Ji X., Branca R.M., Lehtiö J., Guan Z., Filipcik P. (2013). Mammalian Target of Rapamycin (mTor) Mediates Tau Protein Dyshomeostasis: Implication for Alzheimer Disease. J. Biol. Chem..

[16] Sun N., Wang H., Ma L., Lei P., Zhang Q. (2016). Ghrelin attenuates brain injury in septic mice via PI3K/Akt signaling activation. Brain Res Bull..

[17] Long H.Z., Cheng Y., Zhou Z.W., Luo H.Y., Wen D.D., Gao L.C. (2021). PI3K/AKT Signal Pathway: A Target of Natural Products in the Prevention and Treatment of Alzheimer’s Disease and Parkinson’s Disease. Front. Pharmacol..

[18] Kim B., Feldman E.L. (2015). Insulin resistance as a key link for the increased risk of cognitive impairment in the metabolic syndrome. Exp. Amp. Mol. Med..

[19] Vargas E., Podder V., Sepulveda M.A.C. (2021). Physiology, Glucose Transporter Type 4. StatPearls.

[20] Haeusler R.A., McGraw T.E., Accili D. (2018). Metabolic Signalling: Biochemical and cellular properties of insulin receptor signalling. Nat. Rev. Mol. Cell Biol..

[21] Chourpiliadis C., Mohiuddin S.S. (2021). Biochemistry, Gluconeogenesis. StatPearls.

[22] Tangvarasittichai S. (2015). Oxidative stress, insulin resistance, dyslipidemia and type 2 diabetes mellitus. World J. Diabetes..

[23] Bradford Y.M., Toro S., Ramachandran S., Ruzicka L., Howe D.G., Eagle A., Kalita P., Martin R., Moxon S.A.T., Schaper K. (2017). Zebrafish Models of Human Disease: Gaining Insight into Human Disease at ZFIN. ILAR J..

[24] Schlegel A., Gut P. (2015). Metabolic insights from zebrafish genetics, physiology, and chemical biology. Cell Mol. Life Sci..

[25] Marín-Juez R., Jong-Raadsen S., Yang S., Spaink H.P. (2014). Hyperinsulinemia induces insulin resistance and immune suppression via Ptpn6/Shp1 in zebrafish. J. Endocrinol..

[26] Rocha F., Dias J., Engrola S., Gavaia P., Geurden I., Dinis M.T., Panserat S. (2013). Glucose overload in yolk has little effect on the long-term modulation of carbohydrate metabolic genes in zebrafish (Danio rerio). J. Exp. Biol..

[27] Nam Y., Rodriguez I., Shin S., Shim J., Kim N., Kim M., Jeong S., Nuankaew W., Hong B., Kim H. (2021). Characteristics of the New Insulin-Resistant Zebrafish Model. Pharmaceuticals.

[28] Kuwabara S., Yamaki M., Yu H., Itoh M. (2018). Notch signaling regulates the expression of glycolysis-related genes in a context-dependent manner during embryonic development. Biochem. Biophys. Res. Commun..

[29] Tseng Y.C., Chen R.D., Lee J.R., Liu S.T., Lee S.J., Hwang P.P. (2009). Specific expression and regulation of glucose transporters in zebrafish ionocytes. Am. J. Physiol. Regul. Integr. Comp. Physiol..

[30] Zheng P.P., Romme E., Van Der Spek P.J., Dirven C.M.F., Willemsen R., Kros J.M. (2010). Glut1/SLC2A1 is crucial for the development of the blood–brain barrier in vivo. Ann. Neurol..

[31] Ennis K., Felt B., Georgieff M.K., Rao R. (2019). Early Life Iron Deficiency Alters Glucose Transporter-1 Expression in the Adult Rodent Hippocampus. J. Nutr..

[32] Li Y., Chen T., Miao X., Yi X., Wang X., Zhao H., Lee S.M.-Y., Zheng Y. (2017). Zebrafish: A promising in vivo model for assessing the delivery of natural products, fluorescence dyes and drugs across the blood–brain barrier. Pharmacol. Res..

[33] Shen S., Zhang W. (2010). ABC transporters and drug efflux at the blood–brain barrier. Rev. Neurosci..

[34] Abdelilah-Seyfried S. (2010). Claudin-5a in developing zebrafish brain barriers: Another brick in the wall. BioEssays.

[35] Henson H.E., Parupalli C., Ju B., Taylor M.R. (2014). Functional and genetic analysis of choroid plexus development in zebrafish. Front. Neurosci..

[36] O’Brown N.M., Pfau S.J., Gu C. (2018). Bridging barriers: A comparative look at the blood–brain barrier across organisms. Genes Dev..

[37] Carnovali M., Banfi G., Mariotti M. (2019). Zebrafish Models of Human Skeletal Disorders: Embryo and Adult Swimming Together. Biomed. Res. Int..

[38] Meng X.-H., Chen B., Zhang J.-P. (2017). Intracellular insulin and impaired autophagy in a zebrafish model and a cell model of type 2 diabetes. Int. J. Biol. Sci..

[39] Oyelaja-Akinsipo O.B., Dare E.O., Katare D.P. (2020). Protective role of diosgenin against hyperglycaemia-mediated cerebral ischemic brain injury in zebrafish model of type II diabetes mellitus. Heliyon.

[40] Fraher D., Ellis M.K., Morrison S., McGee S.L., Ward A., Walder K., Gibert Y. (2015). Lipid abundance in zebrafish embryos is regulated by complementary actions of the endocannabinoid system and retinoic acid pathway. Endocrinology.

[41] Ka J., Pak B., Han O., Lee S., Jin S.W. (2020). Comparison of transcriptomic changes between zebrafish and mice upon high fat diet reveals evolutionary convergence in lipid metabolism. Biochem. Biophys. Res. Commun..

[42] Huang X., Li Y., Wang T., Liu H., Shi J., Zhang X. (2020). Evaluation of the oxidative stress status in zebrafish (Danio rerio) liver induced by three typical organic uv filters (BP-4, PABA and PBSA). Int. J. Environ. Res. Public Health.

[43] Massarsky A., Kozal J.S., Di Giulio R.T. (2017). Glutathione and zebrafish: Old assays to address a current issue. Chemosphere.

[44] Eames S.C., Kinkel M.D., Rajan S., Prince V.E., Philipson L.H. (2013). Transgenic zebrafish model of the C43G human insulin gene mutation. J. Diabetes Investig..

[45] Lodd E., Wiggenhauser L.M., Morgenstern J., Fleming T.H., Poschet G., Büttner M., Tabler C.T., Wohlfart D.P., Nawroth P.P., Kroll J. (2019). The combination of loss of glyoxalase1 and obesity results in hyperglycemia. JCI Insight.

[46] Zhang Y., Qin C., Yang L., Lu R., Zhao X., Nie G. (2018). A comparative genomics study of carbohydrate/glucose metabolic genes: From fish to mammals. BMC Genom..

[47] van Wijk R.C., Krekels E.H.J., Kantae V., Harms A.C., Hankemeier T., van der Graaf P.H., Spaink H.P. (2019). Impact of post-hatching maturation on the pharmacokinetics of paracetamol in zebrafish larvae. Sci. Rep..

[48] Holt G.J. (2011). Larval Fish Nutrition.

[49] Elo B., Villano C.M., Govorko D., White L.A. (2007). Larval zebrafish as a model for glucose metabolism: Expression of phosphoenolpyruvate carboxykinase as a marker for exposure to anti-diabetic compounds. J. Mol. Endocrinol..

[50] Gut P., Baeza-Raja B., Andersson O., Hasenkamp L., Hsiao J., Hesselson D., Akassoglou K., Verdin E., Hirschey M.D., Stainier D.Y.R. (2013). Whole-organism screening for gluconeogenesis identifies activators of fasting metabolism. Nat. Chem. Biol..

[51] Tanvir Z., Nelson R.F., DeCicco-Skinner K., Connaughton V.P. (2018). One month of hyperglycemia alters spectral responses of the zebrafish photopic electroretinogram. DMM Dis. Model Mech..

[52] Tay S.S., Kuah M.K., Shu-Chien A.C. (2018). Transcriptional activation of zebrafish fads2 promoter and its transient transgene expression in yolk syncytial layer of zebrafish embryos. Sci. Rep..

[53] Kulkarni A.A., Conteh A.M., Sorrell C.A., Mirmira A., Tersey S.A., Mirmira R.G., Linnemann A.K., Anderson R.M. (2018). An in vivo zebrafish model for interrogating ROS-mediated pancreatic β-cell injury, response, and prevention. Oxid. Med. Cell. Longev..

[54] Schultze S.M., Hemmings B.A., Niessen M., Tschopp O. (2012). PI3K/AKT, MAPK and AMPK signalling: Protein kinases in glucose homeostasis. Expert Rev. Mol. Med..

[55] Landgraf K., Schuster S., Meusel A., Garten A., Riemer T., Schleinitz D., Kiess W., Körner A. (2017). Short-term overfeeding of zebrafish with normal or high-fat diet as a model for the development of metabolically healthy versus unhealthy obesity. BMC Physiol..

[56] Xiong X., Song Q., Han C., Gan W., He F., Wei S., Liu H., Xu H. (2016). Insulin Promotes the Expression of the Gluconeogenic Rate-Limiting Enzymes Phosphoenolpyruvate Carboxykinase (Pepck) and Glucose 6-Phosphatase (G6pase) through PI3k/Akt/mTOR Signaling Pathway in Goose Hepatocytes. Braz. J. Poult. Sci..

[57] Ghaddar B., Diotel N. (2022). Zebrafish: A New Promise to Study the Impact of Metabolic Disorders on the Brain. Int. J. Mol. Sci..

[58] Talchai S.C., Accili D. (2015). Legacy effect of foxo1 in pancreatic endocrine progenitors on adult β-cell mass and function. Diabetes.

[59] Al-Masri M., Krishnamurthy M., Li J., Fellows G.F., Dong H.H., Goodyer C.G., Wang R. (2010). Effect of forkhead box O1 (FOXO1) on beta cell development in the human fetal pancreas. Diabetologia..

[60] Qi Y., Zhu Q., Zhang K., Thomas C., Wu Y., Kumar R., Baker K.M., Xu Z., Chen S., Guo S. (2015). Activation of foxo1 by insulin resistance promotes cardiac dysfunction and βmyosin heavy chain gene expression. Circ. Hear Fail..

[61] Liu G.M., Zhang YMTargeting F.B. (2018). Pase is an emerging novel approach for cancer therapy. Cancer Cell Int..

[62] Kratzer I., Vasiljevic A., Rey C., Fevre-Montange M., Saunders N., Strazielle N., Ghersi-Egea J.-F. (2012). Complexity and developmental changes in the expression pattern of claudins at the blood-CSF barrier. Histochem. Cell Biol..

[63] Günzel D., Yu A.S.L. (2013). Claudins and the Modulation of Tight Junction Permeability. Physiol. Rev..

[64] Carvalho L., Heisenberg C.P. (2010). The yolk syncytial layer in early zebrafish development. Trends Cell Biol..

[65] Flannery C., Dufour S., Rabøl R., Shulman G.I., Petersen K.F. (2012). Skeletal Muscle Insulin Resistance Promotes Increased Hepatic De Novo Lipogenesis, Hyperlipidemia, and Hepatic Steatosis in the Elderly. Diabetes.

[66] Takeuchi M., Yamagishi S.-I. (2010). Insulin resistance is an independent correlate of high serum levels of advanced glycation end products (AGEs) and low testosterone in non-diabetic men. Oxid. Med. Cell. Longev..

[67] Hurrle S., Hsu W.H. (2017). The etiology of oxidative stress in insulin resistance. Biomed. J..

[68] Zhang W., Thompson B.J., Hietakangas V., Cohen S.M. (2011). MAPK/ERK Signaling Regulates Insulin Sensitivity to Control Glucose Metabolism in Drosophila. PLoS Genet..

[69] Alvira-Botero X., Carro M.E. (2010). Clearance of Amyloid-β Peptide Across the Choroid Plexus in Alzheimers Disease. Curr. Aging Sci..

[70] Camara A.Y., Wan Y., Yu Y., Wang Q., Wang K., Li H. (2019). Effect of Endogenous Selenium on Arsenic Uptake and Antioxidative Enzymes in As-Exposed Rice Seedlings. Int. J. Environ. Res. Public Health.

[71] Inestrosa N.C., Arenas E. (2010). Emerging roles of Wnts in the adult nervous system. Nat. Rev. Neurosci..

[72] Abdul H.M., Furman J.L., Sama M.A., Mathis D.M., Norris C.M. (2010). NFATs and Alzheimer’s Disease. Mol. Cell Pharmacol..

[73] Killick R., Ribe E.M., Al-Shawi R., Malik B., Hooper C., Fernandes C., Dobson R., Nolan P.M., Lourdusamy A., Furney S. (2014). Clusterin regulates β-amyloid toxicity via Dickkopf-1-driven induction of the wnt–PCP–JNK pathway. Mol. Psychiatry..

[74] Caricasole A., Copani A., Caraci F., Aronica E., Rozemuller A.J., Caruso A., Storto M., Gaviraghi G., Terstappen G.C., Nicoletti F. (2004). Induction of Dickkopf-1, a negative modulator of the Wnt pathway, is associated with neuronal degeneration in Alzheimer’s brain. J. Neurosci..

[75] Mahboudi H., Heidari N.M., Rashidabadi Z.I., Anbarestani A.H., Karimi S., Darestani K.D. (2018). Prospect and Competence of Quantitative Methods via Real-time PCR in a Comparative Manner: An Experimental Review of Current Methods. Open Bioinform. J..

[76] Benjamini Y., Hochberg Y. (1995). Controlling the False Discovery Rate: A Practical and Powerful Approach to Multiple Testing. J R. Stat. Soc. Ser. B Stat. Method..

[77] Young M.D., Wakefield M.J., Smyth G.K., Oshlack A. (2010). Gene ontology analysis for RNA-seq: Accounting for selection bias. Genome Biol..

[78] Keegan B.R., Feldman J.L., Lee D.H., Koos D.S., Ho R.K., Stainier D.Y.R., Yelon D. (2002). The elongation factors Pandora/Spt6 and Foggy/Spt5 promote transcription in the zebrafish embryo. Development.

[79] Sasore T., Kennedy B. (2014). Deciphering combinations of PI3K/AKT/mTOR pathway drugs augmenting anti-angiogenic efficacy in vivo. PLoS ONE.

